# Unravelling soil fungal communities in dumpsites in Kabale, Western Uganda using culturomics

**DOI:** 10.3389/ffunb.2026.1823753

**Published:** 2026-08-06

**Authors:** Adeyinka Odebode, Stephane Ranque

**Affiliations:** 1 Bishop Barham University College, Uganda Christian University, Kabale, Uganda; 2 Aix Marseille University, SSA, APHM, RITMES, Marseille, France; 3 IHU Méditerranée Infection, Aix Marseille University, Marseille, France

**Keywords:** antifungal resistance, culturomics, dumpsites, fungi, public health

## Abstract

Inadequate waste disposal systems have long been a perennial problem, with no clearly safe solution in sight in most developing countries. Dumpsites harbor microbial pathogens in the soil ecosystem that can cause disease outbreaks upon their seepage into the water bodies. However, there is a paucity of investigation on the diversity of fungi in dumpsites in Uganda. Hence, this research aims at evaluating the diversity and distribution patterns of fungi associated with top soils from fifteen different dumpsites in Kabale, South west, Uganda. Culturomics approach was employed in this investigation. Isolation of the fungal species was done using five different culture media, i.e., Potato Dextrose Agar (PDA), Malt Extract Agar (MEA), ChromAgar (CA), FastFung (FF), and Saboraud Dextrose Agar (SDA). Fungal isolation was carried out using the soil dilution plate technique. ITS rRNA gene sequencing was used to identify purified isolates. FF emerged as the most effective agar, yielding the highest number of fungal isolates, followed by PDA, MEA, CA, and SDA. In all, 54 different isolates were obtained. *Trichoderma, Fusarium*, and *Aspergillus* sp were the most prevalent. Also, pathogenic and opportunistic fungi such as *Simplicillium lamellicola, Mortierella multispora*, and *Torulaspora delbrueckii* were isolated. All *Aspergillus* isolates were further screened for azole-resistance (itraconazole, voriconazole, posaconazole, and Amphotericin B). Results showed most *Aspergillus* species have lower MIC; however, *A. clavatus* had an MIC of 1.5 ug/mL. This work further emphasizes the need for regular monitoring of dumpsites for fungi, which can be of public health concern.

## Introduction

Today, waste disposal has become an acute problem in numerous urban centers across the world (Sultana et al., 2021). Of the many problems associated with urbanization, especially in sub-Saharan Africa, the waste management crisis has assumed an important dimension. Many items are discarded every day, ranging from ordinary rubbish to old newspapers, packaging, cleaning materials, and many different kinds of junk (Kwun Omang et al., 2021). The fact that waste levels vary across countries and geographical areas of the world is undeniable. It is estimated that in developing countries, the level of per capita waste will rise significantly with economic development, and developing country municipal solid waste (MSW) streams also exhibit higher organic content than those of developed countries (UNEP, 2018). Solid wastes contribute to health hazards, especially when they are burned freely, covered, or thrown in an unrestricted manner (Odonkor and Salar, 2021). Dumpsite is an old traditional way of waste disposal, similar to the landfill method for managing waste. The microorganisms found in waste dumpsites obtain their nutritional requirements from the waste constituents, thus aiding the detoxification of complex organic molecules (Osazee et al., 2013). It has been estimated that 1.5 million fungal species are present in natural ecosystems, but only 5 –10% have been described formally (Hawksworth, 2001). In line with the estimation of Schmit and Mueller (2007), there are more than 7 million fungal species worldwide. Although the actual number of fungi species remains unknown, only 5-13% of the total estimated global fungal species have been described (Wang et al., 2008). There are several negative environmental effects which could result from improper dumpsite management, such as leachate, foul odor, etc. When waste is dumped on land, microorganisms such as bacteria and fungi proliferate. Most of the microorganisms are potential human pathogens and may cause severe health hazards (Wachukwu, 2010).

Fungi are key actors in soil ecology and emerge as potential bioremediation agents. Yet, information regarding dumpsite fungal diversity and community structure remains relatively scarce, especially in Uganda. This gap is partly due to both extended fungal diversity and limitations of current fungal identification methods. While classical microbiology methods are inadequate in identifying all forms of microbes, metagenomics, which is the current approach used in environmental sciences, is limited by within-fungal species genetic heterogeneity, the relatively high proportion of unidentified nucleotide sequences obtained from fungi that are absent from the reference nucleotide databases, and the detection of nucleotide sequences obtained from “non-viable” species is a clog in the wheel of metagenomics (Elias et al., 2025). Hence, culturomics, a high-throughput method for culturing microorganisms, was employed in this study.

Since the culturomics approach can be extended to the analysis of any ecological niche, we employed this approach to characterize soil fungi community structure in different dumpsites in Kabale, Uganda, to enable us to build novel insights into the identity, distribution, and ecology of dominant dumpsite fungi. This study on the fungal communities associated with dumpsites, particularly in this poorly studied area, is crucial to appraising the beneficial aspects of these soil microbes in degrading trash that litters the environment. Hence, this study was carried out to characterize the diversity of culturable soil fungal communities in dumpsite environments using culturomics and molecular biology, and to evaluate the presence and antifungal susceptibility of opportunistic fungi with potential environmental and public health relevance.

## Materials and methods

### Description of sample site

**Figure 1:**
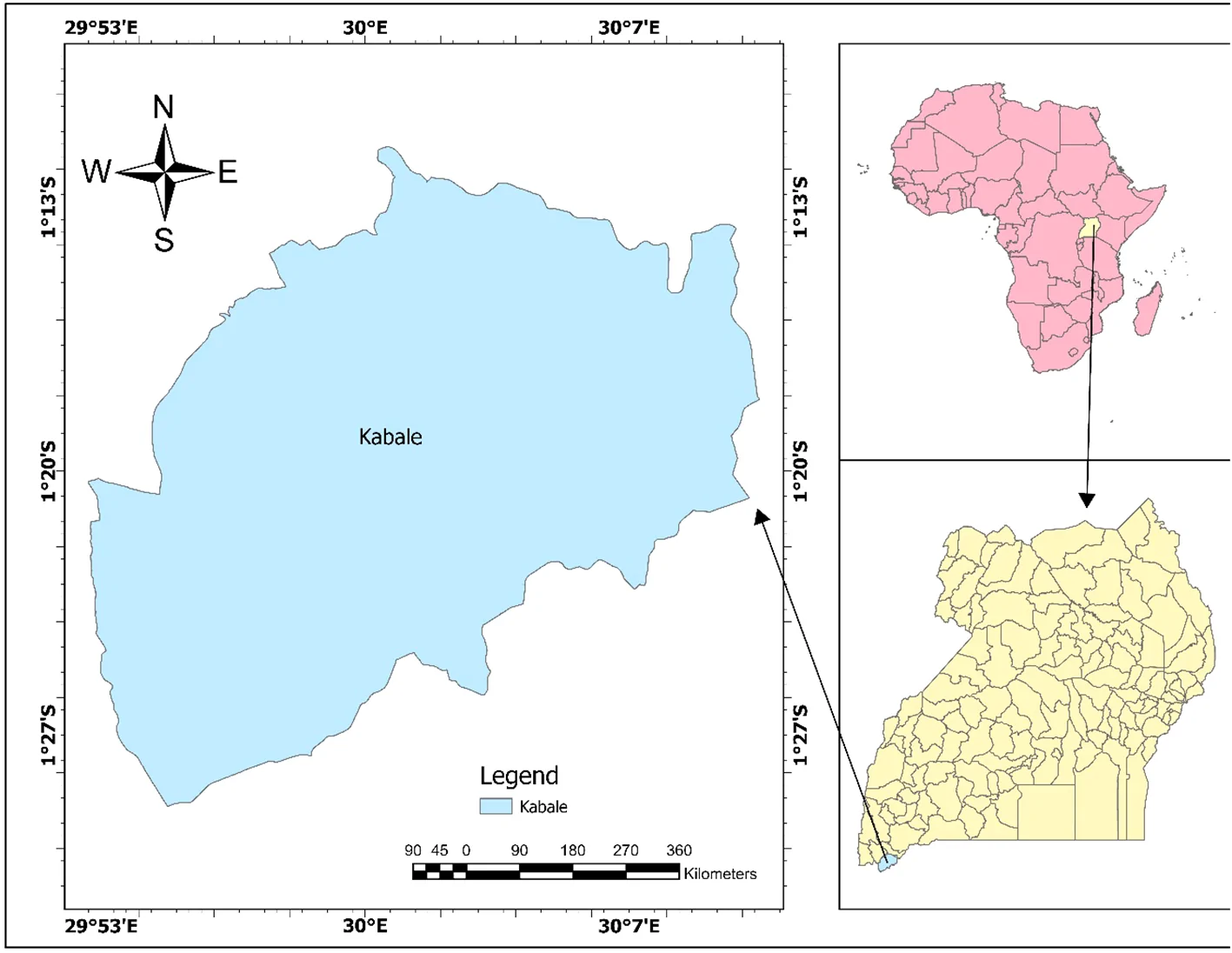
Map showing Kabale town.

The geographical location of the study site is 25°41 26.7” N latitude and 91°55’26.2” E longitude and is located at an elevation of 956 m (**Figure 1**). Kabale is located in the Southwest part of Uganda, close to the Rwanda/Uganda border, called Katuna. The climate of Kabale district is humid sub-tropical with temperatures ranging from 14 °C to 30 °C and an annual precipitation of 2320 mm (https://en.climate-data.org/africa/uganda/western-region/kabale-29827/). Soil texture of the experimental site is silty loam (Clay - 32.58%; Sand - 12.83%; Silt – 54.58%). The study sites consisted of active dumpsites located within mixed agricultural–residential areas, receiving predominantly household and organic waste.

### Sample collection

Soil samples were collected with a soil auger at 0-10cm soil depth, which represents the biologically active zone from fifteen different dumpsites in duplicates, ranging from those primarily of household waste to dumpsites near markets, using a composite sampling approach. Samples from each location were properly labelled and stored in the cold room (-20 °C). Sterile polythene bags were used to transport the collected soil samples to the lab, where they were kept at 4 °C until further analysis.

### Fungal culture and DNA-based identification of the fungal isolates

A total of 5g of soil samples from each duplicate was suspended in 15mL Dulbecco’s Phosphate-Buffered Saline (DPBS). Serial dilutions were prepared for each sample (10^1 to 10^5) and plated onto five different culture media (Sabouraud Dextrose Agar, Potato Dextrose Agar, Chrom agar, Malt Extract Agar, and Fastfung) in replicates. Fastfung media is a new culture medium that can be used for both research and diagnostic purposes to cultivate clinical fungi, even fastidious ones. It is based on Schaedler agar, which has been enhanced with essential elements for the growth of fastidious fungi. Additionally, it has specific antibacterial ingredients for the inhibition of contaminant bacteria growth (Bittar et al., 2021). Media were autoclaved and allowed to cool, after which chloramphenicol was added to inhibit the growth of bacteria in the medium. Plates were incubated at 30 °C and observed after 3 days for growth. Genomic DNA was extracted from the fungal colonies using a bead-beating Fastprep system and appropriate lysis and purification reagents.

The thermal cycler was programmed as follows: an initial denaturation at 95 °C for 15 min, followed by 40 cycles of denaturation at 95 °C for 30s, hybridization at 55 °C for 30 s, and extension at 72 °C for 1:30 s, and a final extension at 72 °C for 7 min.

The fragment of ITS region of the ribosomal unit was amplified with forward and reverse ITS 1F and 4R. PCR amplification was performed using a 25 µL reaction mixture containing 12.5 µL AmpliTaq Gold™ 360 Master Mix (Applied Biosystems, Waltham, MA, USA), 0.75 µL each of forward and reverse primers, 6 µL sterile DNase/RNase-free water, and 5 µL template DNA. Additional primer sets were used selectively for isolates that failed initial amplification. Beta tubulin and Tef genes were also amplified to further confirm *Penicillium* and *Fusarium* isolates.

**Table:** 

ITS1	TCCGTAGGTGAACCTGCGG
ITS 4	TCCTCCGCTTATTGATATGC

The amplified products were purified using a Macherey-Nagel plate (NucleoFast 96 PCR, Düren, Germany). For each amplified fragment, Sanger sequencing was performed in both 5’-3’ and 3’-5’ directions using the same primers used for PCR with BigDye Terminator v1.1, v3.15× sequencing buffer (Applied Biosystems, Warrington, UK) and run on an ABI 3100 automated sequencer (Applied Biosystems). The sequences were assembled using Chromas Pro software, version 1.7.7 (Technelysium Pty. Ltd, Tewantin, Australia), and different alignments were performed with MEGA software. After sequence correction, analysis with Basic Local Alignment Search Tool (BLAST) was performed on the National Centre for Biotechnology Information (NCBI) online database for species identification based on the sequence data. All fungal sequences have been deposited in the NCBI GenBank database (SUB15722719).

A phylogenetic tree was constructed using the maximum likelihood (ML) statistical method. ITS sequences generated in this study were compared with reference sequences obtained from the NCBI GenBank database based on BLAST similarity. Multiple sequence alignment was performed using MUSCLE in MEGA X. Phylogenetic analysis was conducted using the Maximum Likelihood method with the Kimura 2-parameter model. The robustness of the inferred tree was assessed using 1000 bootstrap replicates. The selection of the most appropriate substitution model for constructing the tree was determined by Molecular Evolutionary Genetics Analysis (MEGA) v7.0.26 software.

GraphPad v9.5.1 was used to generate graphs, and Cytoscape v3.10.3 was used to generate agar relationships. Venn diagram showing the comparison between different media used and fungal taxa recovered was constructed using InteractiVenn.

For the antifungal sensitivity test, E-test strips (bioMérieux, Marcy l’Etoile, France) containing different antifungals were tested against selected fungal isolates. Briefly, standardized fungal suspensions were prepared and inoculated onto agar plates, after which E-test strips were applied and incubated. Fungal inocula were prepared to a 0.5 McFarland standard and evenly spread onto agar plates. E-test strips were applied, and plates were incubated at 28–35 °C for 24–72 hours. MIC values were read at the point of intersection between the inhibition ellipse and the strip. Antifungal susceptibility testing was conducted in accordance with standardized procedures consistent with the Clinical and Laboratory Standards Institute guidelines.

## Results

Growth of fifty-four fungal species was checked on Sabouraud Dextrose Agar (SDA), Malt Extract Agar (MEA), Chromagar (CA), FastFung (FF), and Potato Dextrose Agar (PDA). FastFung supported growth in almost all species. Sabouraud Dextrose Agar and Malt Extract Agar supported consistent growth of Aspergilli (*A. awamori, A. clavatus, A. flavus, A. niger, A. oryzae, A. tamari, A. welwitschiae*) as well as some Fusaria (*F. equiseti, F. incarnatum, F. nirenbergiae, F. oxysporum, F. solani*). Chromagar supported only a few taxa: *A. caatinguensis, A. cinerea, C. echinulata, M. alpina, M. circinelloides, P. kudriavzevii*, and *T. koningiopsis*. Potato Dextrose Agar allowed widespread growth with notable representatives in *Trichoderma* (*T. harzanium, T. viride, T. lixii, T. scalesiae), Mortierella (M. alpina, M. multispora)* and *Mucor (M. circinelloides*). Species with limited growth were *A. parasiticus, C. adiposa, T. atroviride*, and *U. vignae*, which grew in one or two media. Hierarchical clustering grouped the *Aspergillus* and *Fusarium* species according to similar consistent growth on Sabouraud Dextrose Agar and Malt Extract Agar, while *Trichoderma* species clustered according to strong growth response on Potato Dextrose Agar and Chromagar (**Table 1**).

**Table 1:** Fungal species identified using different media.

		Media			
Fungal species identified	Saboraud	MEA	Chrome	FastFung	PDA
*Aspergillus flavus*	1	1	1	1	1
*Aspergillus parasiticus*	0	0	0	1	0
*Aspergillus flavipes*	1	1	1	1	1
*Aspergillus tamarii*	0	1	0	1	1
*Aspergillus transmontanensis*	0	0	0	1	0
*Aspergillus clavatus*	1	1	1	1	1
*Aspergillus fumigatus*	0	1	0	1	1
*Aspergillus welwitschiae*	0	1	0	1	1
*Aspergillus toxicarius*	0	1	0	0	1
*Aspergillus awamori*	1	1	0	1	0
*Aspergillus oryzae*	1	1	0	1	1
*Aspergillus niger*	1	1	1	1	1
*Aspergillus texensis*	0	1	1	0	1
*Aspergillus iizukae*	1	0	0	1	1
*Fusarium nirenbergiae*	0	1	1	1	1
*Fusarium solani*	1	1	1	1	1
*Fusarium oxysporum*	1	1	0	1	1
*Fusarium tonkinense*	0	1	1	1	1
*Fusarium incarnatum*	1	1	0	1	1
*Fusarium caeruleum*	0	1	0	1	1
*Fusarium equiseti*	1	1	0	1	1
*Fusarium decemcellulare*	0	0	0	0	0
*Trichoderma atroviride*	0	1	0	0	0
*Trichoderma viride*	1	0	1	1	1
*Trichoderma harzianum*	0	0	0	1	1
*Trichoderma scalesiae*	1	1	1	1	1
*Trichoderma asperellum*	1	0	1	1	1
*Trichoderma koningiopsis*	1	1	1	1	1
*Trichoderma erinaceum*	0	1	0	1	1
*Trichoderma ghanense*	1	0	0	1	0
*Trichoderma lixii*	1	0	0	1	0
*Trichoderma longibrachiatum*	0	1	1	1	1
*Clonostachys farinosa*	0	0	1	1	0
*Chaetomium globosum*	0	0	1	0	1
*Penicillium steckii*	0	1	0	1	1
*Torulaspora delbrueckii*	0	1	1	1	1
*Pichia kudriavzevii*	0	0	1	1	1
*Galactomyces* sp.	0	0	1	1	0
*Geotrichum silvicola*	0	1	1	1	1
*Geotrichum candidum*	0	1	1	1	0
*Mucor circinelloides*	1	1	1	1	1
*Mucor irregularis*	0	0	1	1	0
*Absidia caatinguensis*	0	1	1	1	0
*Absidia cinerea*	0	1	1	1	1
*Mortierella alpina*	1	0	1	1	1
*Mortierella multispora*	1	0	1	1	1
*Mortierella globalpina*	0	0	1	1	0
*Cunninghamella echinulata*	0	0	1	1	1
*Uromyces vignae*	0	0	1	0	0
*Fulvoderma* sp.	1	1	0	1	1
*Simplicillium lamellicola*	1	0	0	1	1
*Ceratocystis adiposa*	0	0	0	1	0
*Sordariomycetes sp*	0	0	1	1	0
*Mortierella globalpina*	0	0	1	1	0

The table above presents a binary (0/1; 1 representing presence and 0 representing absence) matrix showing the growth of 54 fungal species across five culture media: Sabouraud agar, Malt extract agar, Chrome agar, Fastfung media, and Potato dextrose agar (PDA).

### Fungal diversity across culture media

**Figure 2:**
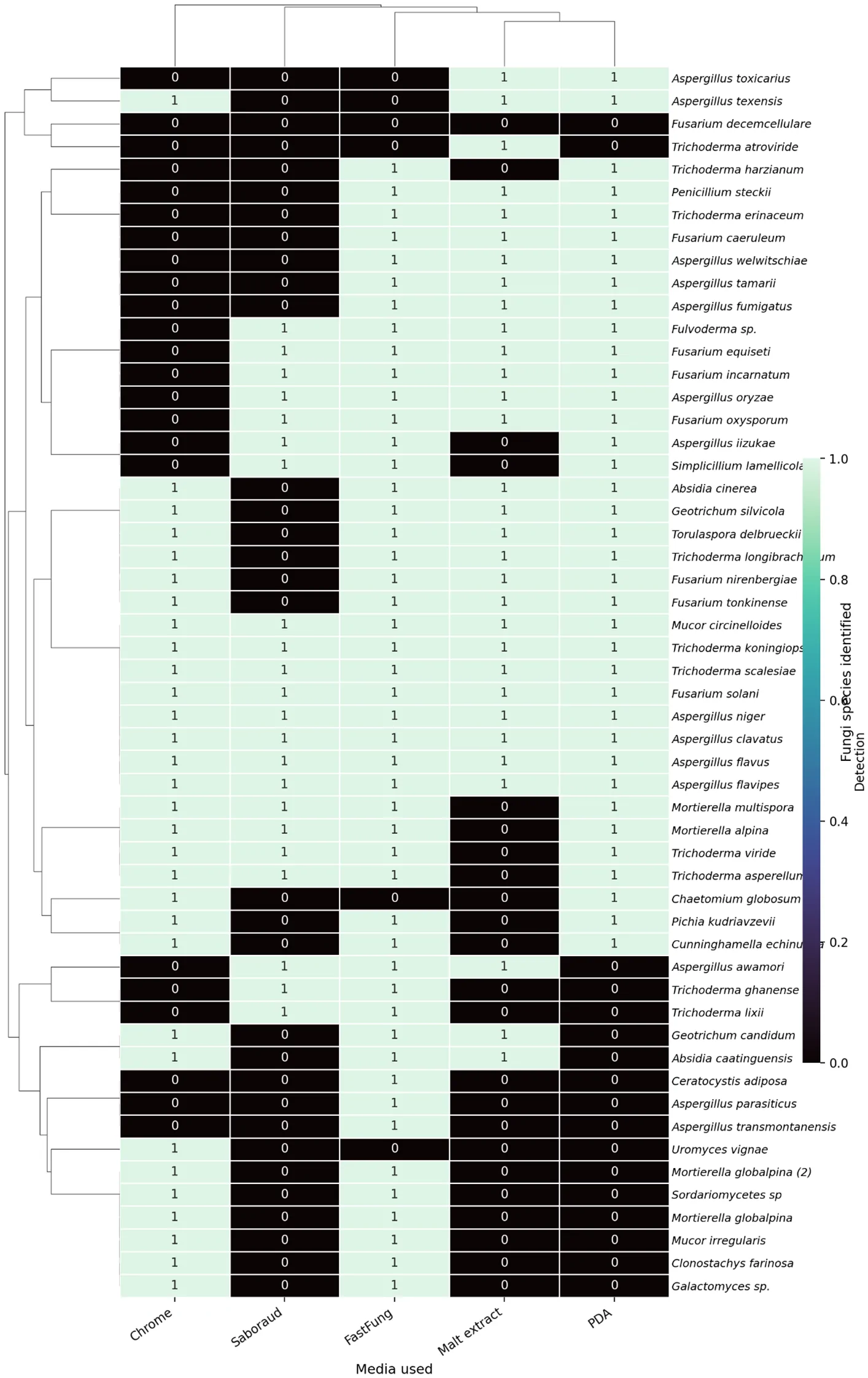
Heat map of fungal species isolated from different media sources. The heat map shows that some species, including *A. flavus*, *A. clavatus*, *A. flavipes*, *A. niger*, *F. nirenbergiae*, *F. solani*, *M. circinelloides*, *T. koningiopsis*, and *T. scalesiae*, grew on all five media. Other species showed media specificity, such as *A. parasiticus* and *C. adiposa*, which only grew on FF medium, while *T. atroviride* grew on MEA. FF media showed the highest success rate in supporting fungal growth. The overall pattern shows that FF successfully identified all fungi species. The FF column shows higher “present” cell counts than other columns across multiple genera, which include *Aspergillus*, *Fusarium* and *Trichoderma*. FF and PDA detected multiple taxa which did not appear on CA. PDA and MEA also achieved good results because they detected many different taxa (**Figure 2**).

**Figure 3:**
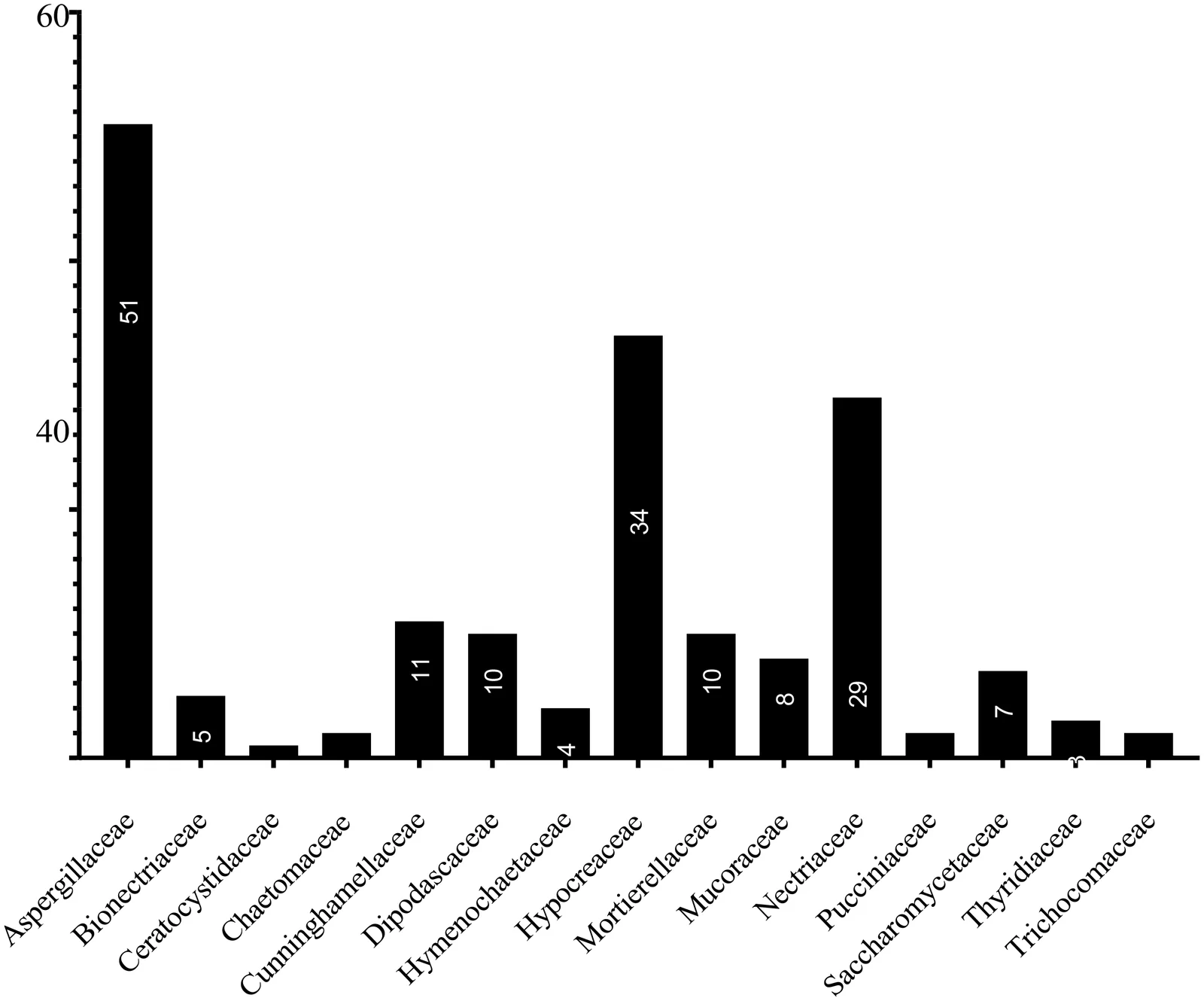
Fungal species isolated according to families. The figure above provides a summary of the overall fungal diversity in terms of family representation, highlighting the dominant families present. *Aspergillaceae*, *Hypocreaceae* and *Nectriaceae* stand out as the most represented families, as opposed to the *Ceratocystidaceae*, *Chaetomaceae*, *Pucciniaceae*, and *Trichocomaceae families*, indicating their prevalence in the dumpsite environment sampled (**Figure 3**). The vertical axis represents number of fungi isolated.

### Community composition and taxonomic distribution

**Figure 4:**
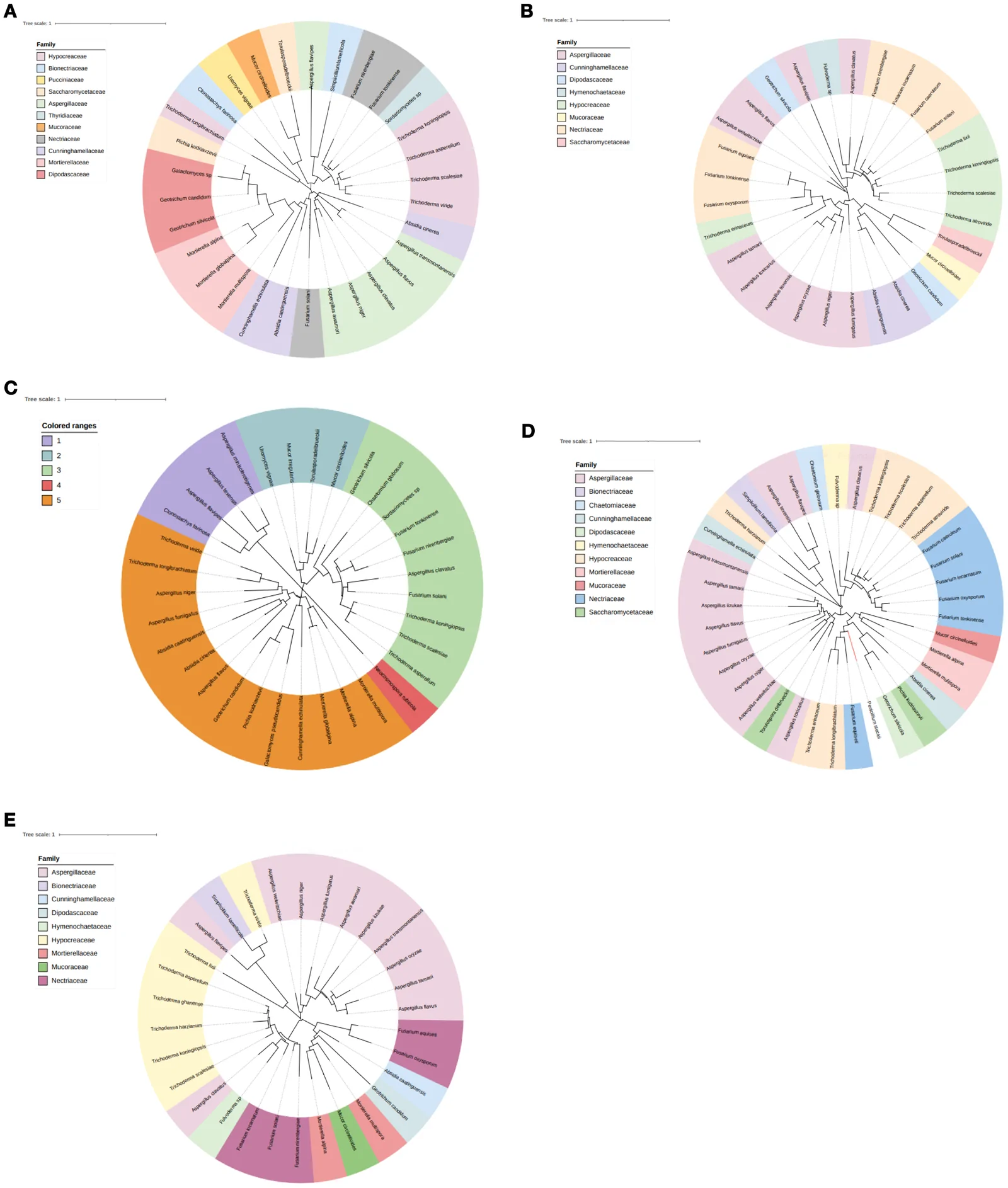
**(A)** Phylogenetic analysis of fungi isolated on FF media. Fungal families within the tree are identified by different color ranges. **(B)** Phylogenetic association of fungi on MEA. **(C)** Phylogenetic association of fungi isolated on CA. **(D)** Phylogenetic relationship of fungi identified on PDA. **(E)** Phylogenetic association of fungi on SDA. Media selectivity and species recovery pattern.

**Figures 4A**–**E** below present circular phylogenetic trees representing evolutionary relationships among fungal species, with each family or clade color-coded accordingly. Each figure shows the distribution of fungal families across the different growth media. The Aspergillaceae, Hypocreaceae, and Nectriaceae families show the highest representation across all media types.

In **Figure 4B**, species are grouped by family, with color distinction as follows: Hypocreaceae, Bionectriaceae, Pucciniaceae, Saccharomycetaceae, Aspergillaceae, Thyridiaceae, Mucoraceae, Nectriaceae, Cunninghamellaceae, Mortierellaceae, and Ophiostomataceae. Overall, species within the same family clustered closely, among which *Aspergillus* species formed a distinct grouping within Aspergillaceae, and *Trichoderma* species grouped under Hypocreaceae.

A parallel phylogenetic tree is shown in **Figure 4C**, but with an alternate color scheme for families represented in the tree, including Cunninghamellaceae, Dipodascaceae, Hymenochaetaceae, and *Saccharomycetaceae*. In this phylogenetic tree, species of *Fusarium* clustered within Nectriaceae, while those of Mucor and Mortierella grouped under Mucoraceae and Mortierellaceae, respectively.

**Figure 5:**
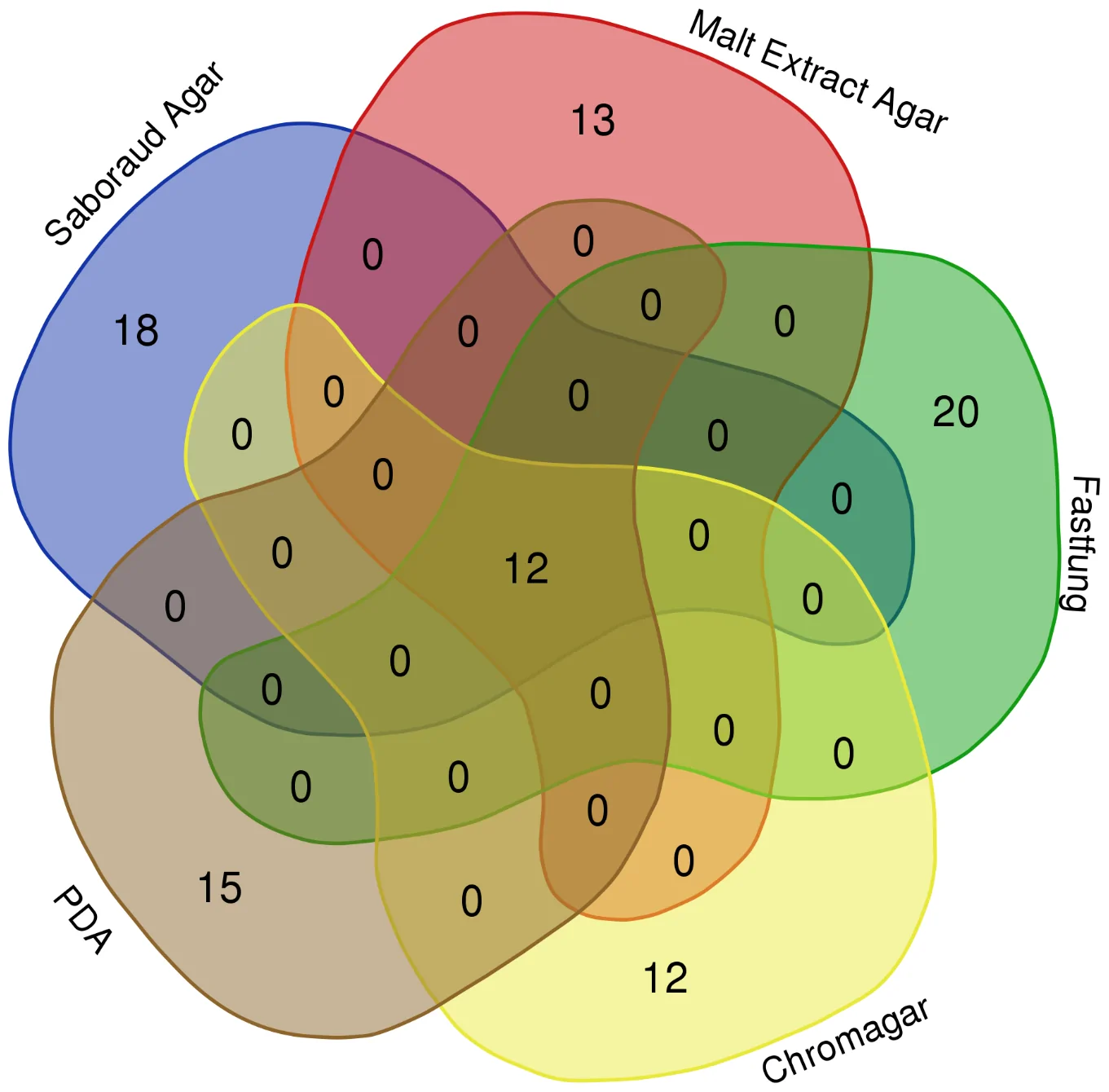
Venn diagram showing the overlap and uniqueness of fungal species recovered across five culture media: Sabouraud Dextrose Agar (SDA), Malt Extract Agar (MEA), Chromagar (CA), FastFung (FF), and Potato Dextrose Agar.

In the tree in **Figure 4D**, clade coloring is divided as follows: Range 1 (Blue): *A. flavus*, *A. oryzae*, *A. parasiticus*, Range 2 (Green): *Fusarium and Trichoderma* species, Range 3 (Red): *Penicillium marneffei*, Range 4 (Orange): *A. niger, A. fumigatus*, *T. viride*, Range 5 (Purple): *A. terreus, A. nidulans, A. versicolor*. The Venn diagram shows that a significant percentage of distinct fungal taxa were recovered from each culture medium, with FF producing the greatest number of species. Strong selectivity is evident from the minimal overlap among media, indicating that media composition significantly impacts fungal recovery (**Figure 5**). Within each range, species showed close genetic relationships, while clade boundaries reflected evolutionary divergences among them.

**Figure 4E** further clarifies family-level clustering using a different color legend. The families represented on this tree are Bionectriaceae, Chaetomiaceae, Didymosphaeriaceae, Hymenochaetaceae, Hypocreaceae, Mortierellaceae, Mucoraceae, and Nectriaceae. The outer ring color-giving thus visually enhances recognition of family-level clusterings, with conserved phylogenetic placement for *Aspergillus*, *Trichoderma*, and *Fusarium* species shown across all trees.

**Figure 6:**
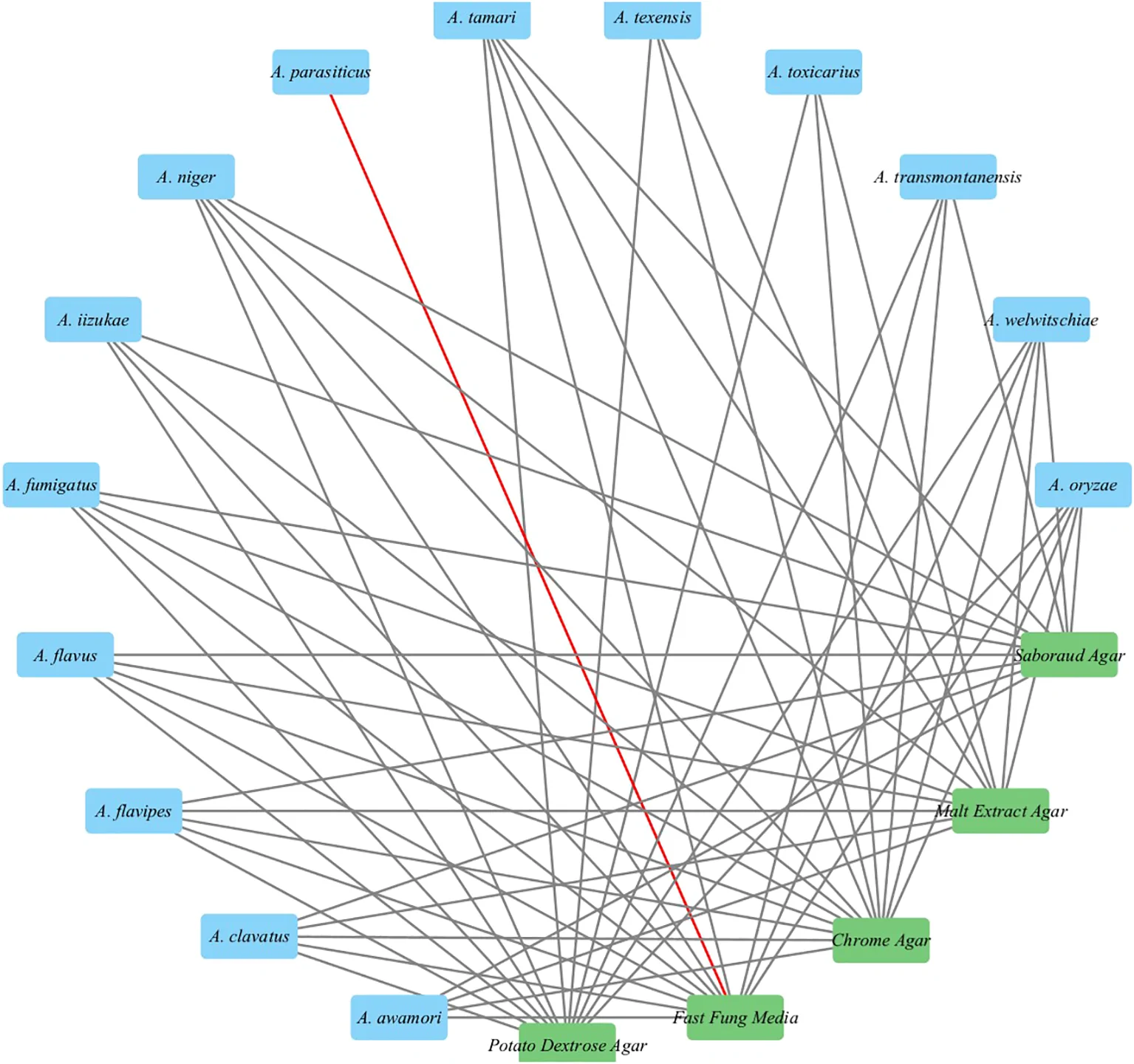
Fungi species identified in the *Aspergillus* family. Bipartite network analysis shows the species within the dominant *Aspergillaceae* family. It shows that some *Aspergillus* species are generalists, growing on all the media (*A. clavatus*, *A. flavipes*, *A. flavus*, *A. niger*), while *A. parasiticus* exhibited a more specialised growth requirement.

Bipartite network analysis shows the species within the dominant *Aspergillaceae* family. It shows that some *Aspergillus* species are generalists, growing on all the media (*A. clavatus, A. flavipes, A. flavus, A. niger*), while *A. parasiticus* exhibited a more specialized growth requirement (**Figure 6**).

**Figure 7:**
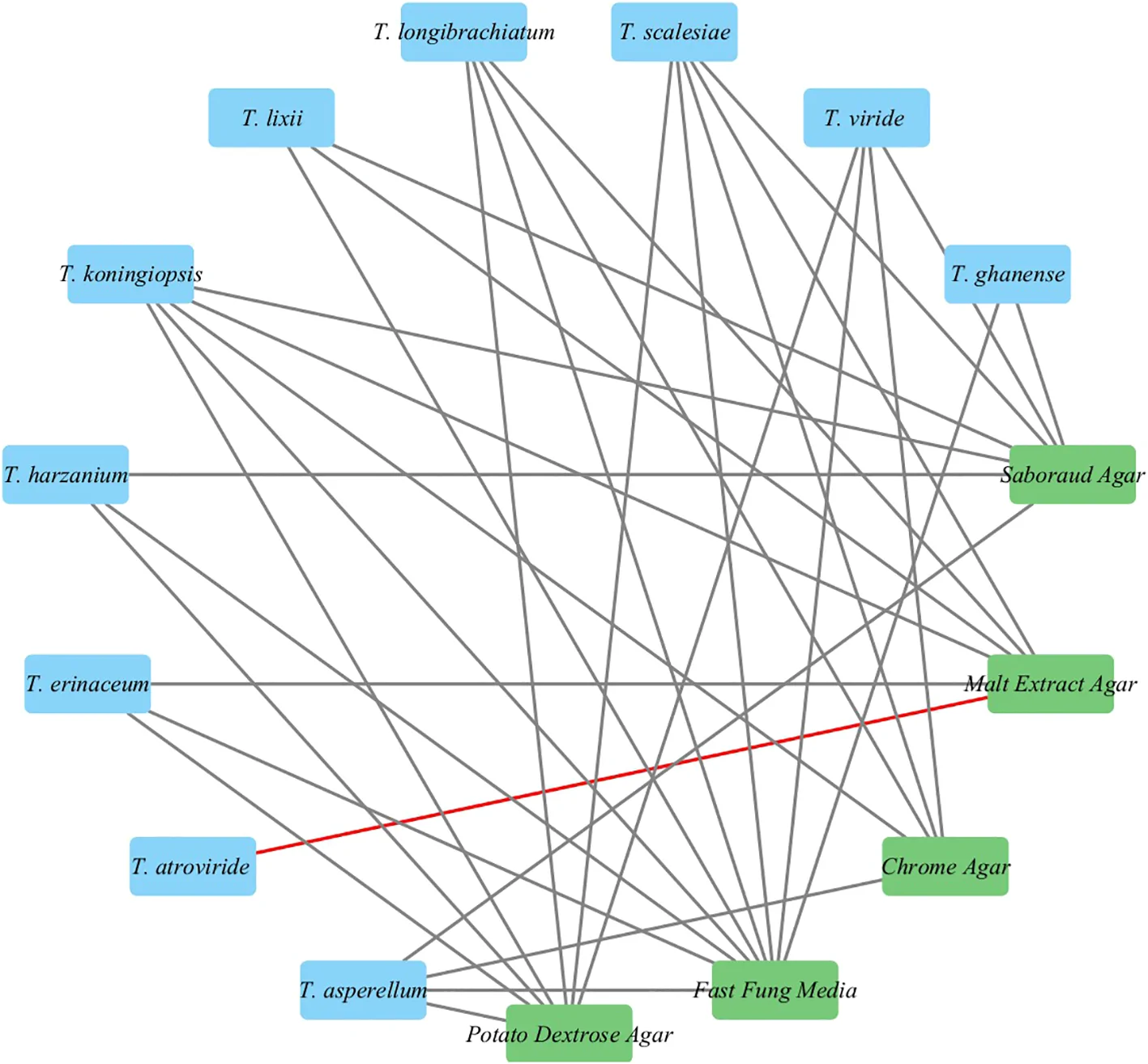
Fungal species identified in the *Hypocreaceae* family. According to the bipartite network analysis, CA demonstrated higher selectivity, whereas FF and PDA supported the greatest number of *Trichoderma species*, suggesting a wide recovery potential. The existence of both media-specific and generalist taxa emphasises how crucial it is to use a variety of culture media in order to fully capture the diversity of fungi.

According to the bipartite network analysis, CA demonstrated higher selectivity, whereas FF and PDA supported the greatest number of *Trichoderma* species, suggesting a wide recovery potential. The existence of both media-specific and generalist taxa emphasizes how crucial it is to use a variety of culture media in order to fully capture the diversity of fungi (**Figure 7**).

The six tested *Aspergillus* species exhibited different sensitivities to common antifungal agents, with *A. parasiticus* showing greater susceptibility to Amphotericin B compared to other antifungals. Among the fungi tested, *A. clavatus* appears the least susceptible isolate overall, because it shows the highest MICs for several drugs, especially caspofungin at 1.5 µg/mL and voriconazole at 0.75 µg/mL. By contrast, *A. flavus* and *A. niger* appear more susceptible to several agents, especially caspofungin, with very low MICs of 0.0094 and 0.016 µg/mL, respectively (**Table 2**).

**Table 2:** Antifungal susceptibility test of selected fungal species against common antifungal agents.

	Antifungals	MIC (µg/mL)		
Fungi	Caspofungin	Posaconazole	Voriconazole	Amphotericin B
*Aspergillus flavipes*	0.25	0.016	0.5	0.25
*A. clavatus*	1.5	0.19	0.75	0
*A. niger*	0.016	0.094	0.19	0
*A. flavus*	0.0094	0.032	0.19	0
*A. fumigatus*	0.064	0.23	0.32	0
*A. parasiticus*	0.032	0.047	0.12	0

## Discussion

Exploring the diversity of dumpsite fungal communities offers valuable insights into which fungi can be explored for bioremediation, which is a better way than burning of refuse, which brings about air pollution and its attendant health implications.

Culture–based approaches are central to isolating and characterizing native strains from contaminated sites, with the aim to further assess their bioremediation potential following a nature-inspired engineering strategy. In this study, a culturomics-based approach combined with ITS barcoding enabled the identification of 54 fungal species from different dumpsite soils in Kabale, providing new insight into fungal diversity in less-studied tropical agroecosystems. Compared to a similar study, our findings revealed a relatively higher diversity. For instance, Khan et al. (2022) reported only 30 species of fungal isolates obtained from municipal solid waste in India. This suggests that dumpsites in Kabale may harbor a richer and more diverse culturable fungal community due to differences in environmental conditions.

Information on the taxonomic and functional profiles of soil fungi in dumpsites can help our understanding of which species can be useful in the biodegradation of certain pollutants in the environment. Consistent with previous reports, our study revealed that *Aspergillaceae*, *Hypocreaceae* and *Nectriaceae* stand out as the most represented families in the different dumpsites sampled. They are frequently associated with organic matter rich environments. Similar observations have also shown that dumpsites could provide a conducive environment for the proliferation of fungi (Kumar et al., 2020).

A key finding of this study is the superior performance of Fastfung medium, which recorded the highest species of fungi compared to other conventional media such as Sabouraud dextrose agar and Potato dextrose agar. This was followed by Saboraud Dextrose agar> Potato dextrose agar> Malt extract agar> CHROMagar. This nutrient-rich composition provides an ideal substrate for fungal proliferation. also confirmed Fastfung’s ability to isolate a variety of fungi. This supports emerging evidence that media composition significantly influences fungal recovery, with enriched media enhancing the isolation of fastidious or slow-growing taxa (Bittar et al., 2021). Such findings underscore the importance of culturomics in reducing culture bias.

The dominant phylum within the soil fungal community in the dumpsites studied was mainly Ascomycetes, Basidiomycetes, and Zygomycetes, which is in agreement with the study done by Shen et al. (2020), where they studied fungi diversity in China. Ascomycota are essential for the cycling of soil nutrients and breaking down organic materials (Sterkenburg et al., 2015). Several member of these taxa are also known to possess biodegradation potential, highlighting their relevance in biotechnological applications. As stated earlier, *Aspergillus* species were the most abundant in all the dumpsites, followed by *Trichoderma* species and *Fusarium* species. Species of Aspergillus are associated with respiratory infections and allergic responses, particularly in immunocompromised individuals (Chen et al., 2023). *Fusarium* species are known producers of mycotoxins and important plant pathogens posing risks to agricultural productivity and human health (Gutiérrez-Sánchez et al., 2023). Some yeasts, such as *Mucor irregularis*, *Rhizopus* sp were also isolated. *Uromyces vignae* was also isolated, which is a rust fungus. The isolation of this fungus shows the consumption of beans as a staple food in Uganda. The occurrence of opportunistic and pathogenic fungi in the dumpsite soils is a reinforcement of the earlier results that dumpsites may serve as reservoirs of medically important fungi. The characteristics of waste disposal sites are high organic matter and microbial activity, which create favorable conditions in the context of proliferation of opportunistic pathogens, such as *Aspergillus*, *Fusarium*, and other airborne fungi (Ayangbenro and Babalola, 2020; Ezeokoli et al., 2023). Research has also shown that contact with bioaerosols from waste sites, especially fungal spores, can also lead to respiratory diseases like allergies, asthma, and invasive infections, particularly in vulnerable populations (Cyprowski et al., 2021; Ghosh et al., 2022).

These results emphasize the role of environmental monitoring, correct waste management measures, and microbial risk assessment to reduce exposures to harmful fungal pathogens. For instance, members of the genera *Fusarium, Aspergillus* and *Penicillium* comprise several species of opportunistic potential plant pathogens affecting agriculture and horticulture globally, and have increasingly been found to also affect humans. A study by Oyedepo et al. (2019) similarly reported that dumpsites are a breeding ground for pathogenic fungi, which could lead to respiratory problems and other issues. Many fungi are opportunistic organisms and cause infections in individuals undergoing extended antibiotic treatment or experiencing severe immunosuppression. For instance, *Aspergillus* species are well recognized opportunistic fungi associated with respiratory infections (WHO, 2002). *Aspergillus* species prevalence in landfills may be explained by their widespread distribution. They can colonize a wide range of substrates. The high abundance of *Aspergillus* species in this study is consistent with earlier findings that these organisms are extensively dispersed and capable of colonizing a variety of substrates (Singh et al., 2020; Zhang et al., 2020). The spores of *Aspergillus* in the environment have been reported to cause allergic response and opportunistic infection in immunocompromised persons (Bennet, 2010). The abundance of Aspergillus species that was observed in our study is in line with findings that indicate their ubiquitous distribution and ecological adaptability in both natural and anthropogenically-disturbed environments (Houbraken et al., 2020; Vesth et al., 2021). These fungi have been well adapted to nutrient-rich and stress-prone habitats, allowing them to colonize a wide variety of substrates, including contaminated soils and waste-associated environments. Nonetheless, their prevalence in the environment also raises health concerns among people. Recent world estimates further emphasize the increased burden of aspergillosis, particularly in the low- and middle-income environments where exposure risks are likely to be heightened.

In agreement with our study, several other studies have reported diversity of fungi in dumpsites and landfills. The most common ones are *Aspergillus*, *Penicillium*, *Fusarium*, *Alternaria*, *Trichoderma*, *Curvularia*, *Rhizopus*, *Geotrichum* and *Acremonium*, many of which are linked to the decomposition of organic matter and opportunistic pathogenicity. Several studies also confirm that dumpsites contain not only saprophytic but also potentially pathogenic taxa, which are influenced by substrate composition, moisture, and other environmental conditions (Ezeokoli et al., 2023; Ghosh et al., 2022; Ayangbenro and Babalola, 2020). These findings are consistent with the genera identified in our study and suggest a common pattern in the fungi diversity associated with solid waste disposal sites. The presence of potentially pathogenic fungi highlights the need for mycological surveillance of environments, particularly near dumpsites. At the same time, beneficial taxa such as *Trichoderma* species present in dumpsite soil can detoxify toxic compounds and accelerate the degradation of organic material (Zin and Badaluddin, 2020). Apart from the beneficial species, the genus *Trichoderma* also comprises species which are highly dangerous to human health (Druzhinina et al., 2008). They constitute a lethal hazard for individuals with reduced resistance, including patients with leukemia, HIV positive or having transplants (Kredics et al., 2003). Infections caused by *Trichoderma* are typically diagnosed late and are difficult to treat, as these fungi exhibit a low sensitivity to commonly applied antifungal drugs (Kratzer et al., 2006) and combined treatment is frequently necessary (Alanio et al., 2008). Larsen et al. (1998) reported that *Trichoderma* spp. may cause diseases of the respiratory tract in humans due to the volatile metabolites they produce. *Fusarium* species, which are the third most predominant fungal species in Kabale dumpsites, are known to produce three important classes of mycotoxins, namely trichothecenes, fumonisins, and zearalenones with their mycoestrogens. These toxins are highly toxic and carcinogenic to farm and laboratory animals and have been associated with human Esophageal cancer and birth defects (Leslie and Summerell, 2006; Ekwomadu and Mwanza, 2015). Aside from its implications in human pathology, *Fusarium* spp. is an important genus due to its pathogenicity against plants, as it causes a complex disease to manage and is a very common threat to maize, wheat and other grain plants (Batista et al., 2003).

Even though in this study antifungal susceptibility testing indicated that there was no resistance in the tested *Aspergillus* isolates, all values were below the threshold value for resistance. The greater problem of environmental antifungal resistance is becoming an increasing concern. There has been an increasing association between environmental exposure to agricultural fungicides and pharmaceutical residues and the emergence and spread of antifungal-resistant fungi. The widespread use of azole fungicides in agriculture, in particular, has been implicated in the development of cross-resistance in clinically relevant fungi such as *Aspergillus fumigatus*, which is raising significant public health concerns (Fisher et al., 2022; Fraaije et al., 2020). These results highlight the importance of continuous environmental monitoring and integrated surveillance strategies, particularly in waste dumping sites.

Dumpsites create a good environment for the growth and spread of various fungal communities, including opportunistic as well as pathogenic species. These microorganisms can be transported through soil profiles to under groundwater, increasing the likelihood of the contamination of aquifers. Studies have revealed that waste site leachate can transport microbial contaminants, such as fungi, into the surrounding water bodies, potentially causing health hazards to the local populations (Ezeokoli et al., 2023). When contaminated water is utilized for domestic purposes, such as drinking, cooking and washing, it can be a source of exposure pathways that promote at-risk infections, especially among vulnerable populations. These findings underscore the importance of better waste management strategies and frequent monitoring of the water quality in places around the dumpsites.

Taken together, our findings provide baseline data on diversity, distribution, and potential functional roles of culturable fungal communities in dumpsite soils in Kabale. Future studies integrating culture-independent methods will be essential to fully characterize these communities. However, our discoveries highlight the ecological and public health relevance of fungi in dumpsite settings.

## Conclusion

This study has shown that culturomics is a robust method to reveal a broad and underexplored fungi in dumpsites. This method is a more comprehensive method compared to traditional methods of culturing fungi. The culturomics method revealed rare, opportunistic, and industrially relevant fungi found in dumpsites. Our findings show that integrating culturomics with molecular biology can be used for environmental monitoring to predict health hazards associated with certain fungi, which could serve as a public health concern.

## Data Availability

The datasets presented in this study can be found in online repositories. The names of the repository/repositories and accession number(s) can be found in the article/**Supplementary Material**.
