# Supplementary material for: Unravelling soil fungal communities in dumpsites in Kabale, Western Uganda using culturomics
Source: Front Fungal Biol. 2026 Aug 6;7:1823753. doi: 10.3389/ffunb.2026.1823753 (PMC13490154; doi:10.3389/ffunb.2026.1823753)
Supplement: Supplementary file 1 [file SupplementaryFile2.pdf]

Isolate ID	Identified species	% identity	Query cover
KD 01	*Aspergillus flavus*	92.90%	94%
KD 02	*Aspergillus parasiticus*	95.76%	96%
KD 03	*Aspergillus flavipes*	93.20%	95%
KD 04	*Aspergillus tamarii*	94.50%	89%
KD 05	*Aspergillus transmontanensis*	99%	87%
KD 06	*Aspergillus clavatus*	97.85%	99.00%
KD 07	*Aspergillus fumigatus*	96.70%	86%
KD 08	*Aspergillus welwitschiae*	93.20%	91%
KD 09	*Aspergillus toxicarius*	94.30%	90%
KD 10	*Aspergillus awamori*	91.60%	89%
KD 11	*Aspergillus oryzae*	92.16%	91%
KD 12	*Aspergillus niger*	93%	51%
KD 13	*Aspergillus texensis*	90.80%	68%
KD 14	*Aspergillus iizukae*	95.77%	69%
KD 15	*Fusarium nirenbergiae*	92.10%	79%
KD 16	*Fusarium solani*	89.90%	67%
KD 17	*Fusarium oxysporum*	90.10%	79%
KD 18	*Fusarium tonkinense*	95.50%	83%
KD 19	*Fusarium incarnatum*	92.20%	88%
KD 20	*Fusarium caeruleum*	91.90%	94%
KD 21	*Fusarium equiseti*	98.31%	88%
KD 22	*Fusarium decemcellulare*	89.10%	100%
KD 23	*Trichoderma atroviride*	96.58%	88%
KD 24	*Trichoderma viride*	94.29%	100%
KD 25	*Trichoderma harzianum*	90.10%	76%
KD 26	*Trichoderma scalesiae*	92.89%	98%
KD 27	*Trichoderma asperellum*	95.60%	89%
KD 28	*Trichoderma koningiopsis*	94.34%	67%
KD 29	*Trichoderma erinaceum*	93.87%	89%
KD 30	*Trichoderma ghanense*	94.67%	87%
KD 31	*Trichoderma lixii*	98.65%	78%
KD 32	*Trichoderma longibrachiatum*	92.89%	89%
KD 33	*Clonostachys farinosa*	93%	91%
KD 34	*Chaetomium globosum*	90.80%	90%
KD 35	*Penicillium steckii*	95.77%	88%
KD 36	*Torulaspora delbrueckii*	94.54%	89%
KD 37	*Pichia kudriavzevii*	0.9834	66%
KD 38	*Galactomyces sp.*	89.90%	69%
KD 39	*Geotrichum silvicola*	90.10%	88%
KD 40	*Geotrichum candidum*	93.10%	67%
KD 41	*Mucor circinelloides*	92.20%	79%
KD 42	*Mucor irregularis*	91.90%	78%

KD 43	*Absidia caatinguensis*	98.31%	81%
KD 44	*Absidia cinerea*	89.10%	90%
KD 45	*Mortierella alpina*	90.80%	84%
KD 46	*Mortierella multispora*	93.50%	89%
KD 47	*Mortierella globalpina*	98.10%	87%
KD 48	*Cunninghamella echinulata*	89.68%	90%
KD 49	*Uromyces vignae*	90.50%	60%
KD 50	*Fulvoderma sp.*	96.10%	87%
KD 51	*Simplicillium lamellicola*	94.20%	88%
KD 52	*Ceratocystis adiposa*	90.78%	87%
KD 53	*Sordariomycetes sp*	93.69%	65%
KD 54	*Mortierella globalpina*	94%	78%
