# Supplementary material for: Unravelling soil fungal communities in dumpsites in Kabale, Western Uganda using culturomics
Source: Front Fungal Biol. 2026 Aug 6;7:1823753. doi: 10.3389/ffunb.2026.1823753 (PMC13490154; doi:10.3389/ffunb.2026.1823753)
Supplement: Supplementary file 2 [file SupplementaryFile1.docx]

>seq1

TTTCTAATTTGGGTGTTTTACGGACGTGGATGCGCCGCGCTCCCGGTGCGAGTTGTGCGA

ACTACTGCGCAGGAAACGCTGCGGCTAGTTCGCGTATGCCTTTCGGGGCCGGTATCCCGT

CTTACGGGCTCCCGAGGTCCCCAACGCCGATCCCCCTTTGGAGGGGTTCGAGGGTTTGAA

ATGACGCTCGGACAAGCATGCCCGCCAGAATACTGGCGGGCGCAATGTGCGTTCAAAGAT

TCGATGATTCACTGAATTCTGCAATTCACATTACTTATCGCATTTCGCTGCGTTCTTCAT

CGATGCA

>seq2

TTTCAAATTTGGGTGCTTTACGGGCGTGGCCGCGCCTGCTCGCCGGTGCGAGTTGTGCCG

ACTACTGCGCAGGAGAGGCTGCGGCGAGACCGCGACTGTATTTCGGGGCCGGGATCCCGT

CTTAGGGGCTCCCGAGGTCCCCAACGCCGACCCCCCGGAGGGGTTCGAGGGTGTGAAATG

ACGCTCGGACAGGCATGCCCGCAAGAATACTGGCGGGCGCAATGTGCGTTCAAAGATTCG

ATGATTCACTGAATTCTGCAATTCACATTACTTATCGCATTTCGCTGCGTTCTTCATCGA

TGC

>seq3

GCGTGGCCGCGAGATTACCATAAAGAGGGTTTTACTACTACGCTATGGAAGCTCGACGTG

ACCGCCAATCAATTTGAGGAACGCGAATTAACGCGAGTCCCAACACCAAGCTGTGCTTGA

GGGTTGAAATGACGCTCGAACAGGCATGCCCGCCAGAATACTGGCGGGCGCAATGTGCGT

TCAAAGATTCGATGATTCACTGAATTCTGCAATTCACATTACTTATCGCATTTTGCTGCG

TTCTTCATCGATGCA

>seq4

GTAGGGTTCCTAGCGAGCCCAACCTCCCACCCGTGTTTACTGTACCTTAGTTGCTTCGGC

GGGCCCGCCATTCATGGCCGCCGGGGGCTCTCAGCCCCGGGCCCGCGCCCGCCGGAGACA

CCACGAACTCTGTCTGATCTAGTGAAGTCTGAGTTGATTGTATCGCAATCAGTTAAAACT

TTCAACAATGGATCTCTTGGTTCCGGCATCGATGAAGAACGCAACAAGATGAACAAACGG

GGCATCAACGACTCAATCCATTCACCGCCTTCCCCATTTCACACTAGTTATCGCATTTCG

CTGCTTTCTTCATCGATGCATATACCCCGCCGGGGACCAAAAAAAGGGGGGGAAATTGGA

AAAAAGGGAGGGAATTGGGGTTGTTTTTTTAAAAACTAAAAAAAAAAAAATTT

>seq5

AATCCGTGTTTTTATGGACTTTAAAAATCCATATCCACCTTGTGTGCAATGTCATCTCTC

TGGGGGCTGCCGGCTGTCAAAAGCCGTGTGGTCACCTTTGGGATTTATATCTACTCAGAA

CTTTAGTGATTTTGTCTGAAACATATTATGAATACTTAATTCAAAATACAACTTTCAACA

ACGGATCTCTTGGCTCTCGCATCGATGAAGAACGCAGCATCGATGA

>seq6

CCTGACCGAGTCAAGAAAAAAATTGGGTGTTTAACGGCTGTGGACGCGCCGCGCCCCCGA

TGCGAGTGTGCAAACTACTGCGCAGGAGAGGCTGCGGCGAGACCGCCACTGTATTTCGGA

GACGGCCACCCGCTAAGGGAGGGCCGATCCCCAACGCCGACCCCCCGGAGGGGTTCGAGG

GTTGAAATGACGCTCGGACAGGCATGCCCGCCAGAATACTGGCGGGCGCAATGTGCGTTC

AAAGATTCGATGATTCACTGAATTCTGCAATTCACATTACTTATCGCATTTCGCTGCGTT

CTTCATCGATGC

>seq7

CCGTACCACTGTTGCTTCGGCGGGCCCGCCAGCGTCCGCTGGCCGCCGGGGGGCTTCTGC

CCCCGGGCCCGTGCCCGCCGGAGACCCCAACACGAACACTGTTTCTGAAAGCCTGTATGA

ATCCGATTCTTTGTAATCAGTTAAAACTTTCAACAATGGATCTCTTGGTTCCGGCATCGA

TGAAGAAACGCAGCATATGAAGAAAGCGGCATCGATGAAGAACGCCCCCCCCATCAAACC

ACGCCCCTTTTTTTTTTTGGGGGGCCCCCCCCTCTTTTGGGGGGGGGGGTTTGCCCGCAC

CCGGGGCCAAAAAAAAAAAAAAAAAGGCTGCGCCTTTTTTTTAAAACACTTTTGTTCAGA

GGGTATAATTTCTTACAAAAATTTTTTGTGTGCGCGCCCCAAAAAAA

>seq8

GTAGGGTTCCTAGCGAGCCCAACCTCCCACCCGTGTTTACTGTAACCTTAGTTGCTTCGG

CGGGCCCGCCTTTAAGGCCGCCGGGGGGCATCAGCCCCCGGGCCCGCGCCCGCCGGAGAC

ACCACGAACTCTGTCTGATCTAGTGAAGTCTGAGTTGATTGTATCGCAATCAGTTAAAAC

TTTCAACAATGGATCTCTTGGTTCCGGCATCGATGAACAACGCAGCAAATGTGCGAAGCG

GCACAAGCAAACCCAACCTCCCCCCGGGTTTTCTGTAACCCTAGCGCTTCCGCGGGCCCG

CCTTTAAGGCCCCCCGGGGCGCATCCCCCCCCGGGCCCGGGGCGGGGGGGGGGAAAACCA

CAAACCCCGGCTAAACTGAAGAAATTCTGAATTAATTGTATCCCAATTCAATAAAAACTT

TTAAGGGTTGCGGCCCCTATAAAA

>seq12

CGGAAATACCGATGTGGTTCCTACCCGCGTGACTCTTCCCGTGTGTACTGCCCTTAGTTG

CTTCGGCGGGGTGCGTTTCTGGCCGCAGGGGGCTCCCGCCCGAGGCCCGCCCCAACTCTT

TATGTCTACACCTTCGCGGGATCTAAGAATCCGAACCTGATTGGCGCCCAATCTAGCGTT

TCGAAAATGAGGCTCTCTTGGTTCCGGCGATCTCTTGGTTCTGGCACATGAA

GAAAAAACAAGTCGGGGGGTTTTTTACCCTCCTAATCTTCTATTCTGTGCACCGTTTTTT

TGTGGGGGGTGCTTCGGTACCCCCTCAATCTGGGTATGTTCACTTTTTCCTTTGGGAGAT

TGTTTGGCTGCCCAGATGATTTATTATACTATTATGACTGAACCATAACAGAAAATTGTT

AACATATAAACAACTTTCAGCAATGGATCTCTCGGCTTTCGCATCGATGAAGAACGCACA

>seq13

ACCTATATTGTGGGGTCGCTTCAAATGCGTTTATACCTTCTCGTCCCWTCGTGGGTCTGA

CCCATGCGCAATGTTTTTTTTAAGGGGTATTCTTTTGGGGGTACTCGTTTTAATTTCTTA

TGGTTGTTTTTTGGCCCTGATTATTAATTTTTTTAAACTAAAATTACTGAATAACGATTG

ACCGGTTCTTATGGTTGTTAAAAAATATATTAATTTATATAAAAACAACTTTCAGCAATG

GATCTCTCGGCTTTCGCATCGATGAAAACGCAGCA

>seq14

GGAAATATATGTCGGGGGGTTTTCCCCCTCAAAATCTTTTTTCTGTGCCCGTTTTTTTGT

GGGGGGTGTTCGGTACCCCCTCAATCTGGGTATGTTCACTTTTTCCTTTGGGAATTGTTT

GGCTGCCCTCAGAATTTATATACTATTATGACTGAACTTTACCAAAAATTGTTAACATAT

AAACAACTTTCACAATGGATCTCTCGGCTTTCGCATCGATGAAGAACGCAC

>seq15

TTTACAACCTCCCAACCCCTGTGAACATACCTATACGTTGCCTCGGCGGATCAGCCCGCG

CCCTGTAAAAAGGGACGGCCCGCCCGAGGACCCTAAACTCTGTTTTTAGTGGAACTTCTG

AGTAAAACAAACAAATAAATCAAAACTTTCAACAACGGATCTCTTGGTTCTGGCATCGAT

GAAGAACGCAGCAAAATGCGATAAGTAATGTGAATTGCAGAATTCAGTGAATCATCGAAT

CTTTGAACGCACATTGCGCCCGCCAGTATTCTGGCGGGCATGCCTGTTCGAGCGTCATTT

CAACCCTCAAGCTCAGCTTGGTGTTGGGACTCGCGGTAACCCGCGTTCCCCAAATCGATT

GGCGGTCACGTCGAGCTTCCATAGCGTAGTAATCATACACCTCGTTACTGGTAATCGTCG

CGGCCACGCCGTAAAACCCCAATTTCTYATGTACCGGATCAGGTAGGA

>seq16

TCCGCTTATTGATATGCTTAAGTTCAGCGGGTATTCCTACCTGATCCGAGGTCAACATTT

CAGCAAAGTTGGGTGTTTTACGGACGTGGACGCGCCGCGCTCCCGGTGCGAGTTGTGCAA

ACTACTGCGCAGGAGAGGCTGCGGCGAGACCGCCACTGTATTTCGGGGCCGGCACCCGTG

TGAGGGGTCCCGATCCCCAACGCCGATCCCCCGGAGGGGTTCGAGGGTTGAAATGACGCT

CGGACAGGCATGCCCGCCAGAATACTGGCGGGCGCAATGTGCGTTCAAAGGATTCGATGA

TTCAGCTGAATTCTGCAATTGCACATTACTTATCGCATTTCGCTGCGTTCTTCATCGATG

CA

>seq17

TCCTACCTGTCGAGGTACATTAGGAATGGGGGTTTTACGGCTTGGCCGCGCCGCTCTCCA

GTAAGCAAGGTGTTAGCTACTACGCAATGGAAGCTGCGGCGGGACCGCCACTGTATTTAG

GGGACGGCGTATGCCCACAGGGGGCTTTCCGCCGATCCCCAACGCCAGGCCCGGGGGCCT

GAGGGTTGTAATGACGCTCGAACAGGCATGCCCGCCAGAATACTGGCGGGCGCAATGTGC

GTTCAAAGATTCGATGGTTCACTGAATTCTGCAATTCACATTACTTATCGCATTTCGCTG

CGTTCTTCATCGATGC

>seq18

GCCTCAGCGGATGCTCTGCCCCGGGTGCGTCGAAGCCCCGGTGCAAGGCGCCCGCCGGAG

AGCTGCGGCAATCTTTTTGTTGACCCCCTCACAGGTTTTTTATAATCTGAACGTTCTAGG

CCCCTCCCGTCCCCGTTTCGGAGTTGAAGGGTTGAAATGACGCTGGATCGGCTGGCTCGC

GCAAAATACTGGCAGGCGCACTGTGCGTTCAAAGATTCGATGATTCACTGAATTCTGCAA

TTCACATTACTTATCGCATTTCGCTGCGTTCTTCATCGATGCAGCGTTCTTCATCGATGC

AGCGTTCTTCATCGATGCTGCGTTCTTCATCGATGCTGCGTTCTTCAT

>seq19

TGACAGTTTTAACAGATTACAAAGAATGGATTTTACACGCTGTTAGAAACGTGTTTTTAT

GACTATAAAAAGAGGGGGCCCGGGCAGAAGCCCCCCGGCGGCCAGCGGACGCGGAGGCCC

GCGAAGCAACAAGGTACAGTAGTCACGGAGGTGGTTTGAATCATGGACCCTCTCTCCTCA

CATTTGGCTACGTGAAATGCGGAAGGATCATTACCGAGTGAGGGTCCTCGTGGCCCAACC

TCCCACCCGTGACTACTGTACCACTGTTGCTTCGGCGGGCCCGCCAGCGTCCGCTGGCCG

CCGGGGGGCTTCTGCCCCCGGGCCCGTGCCCGCCGGAGACCCCAACACGAACACTGTTTC

TGAAAGCCTGTATGAATCCGATTCTTTGTAATCAGTTAAAACTTTCAACAATGGATCTCT

TGGTTCCGGCTCGATGAACGACGCACATTGTGCGTTCAAAGACTCGATGATTCAAAGAAT

TCTGCCTTCACAATAKTTATCGCATTTTGCTGCGTTCTTCATCGATGCGGAACCAGTATA

CCGTTGTTGAATTTTAAGTGAGGGGTGAAATCGGCCCCTCCCCCCCCCCGGAGGAACGTT

>seq20

AAGCCAAAACTAGGATTATTTATTGATTTTTTCCGTAGGTGAACCTGCGGAAGGATCATT

AAATAATCAATAATTTTGGCTTGTCATTATTATCTATTTACTGTGAAATGTATTATTACT

TGACGCTTGAGGGATGCTCCACTGCTATAAGGATAGGCGGTGGGGATGCTAACCGAGTCA

TAATCAAGCTTAGGCTTGGTATCCTATTATTATTTACCAAAAGAATTCAGAATTAATATT

GTAACATAGACCTAAAAAATCTATAAAACAACTTTTAACAACGGATCTCTTGGTTCTCGC

ATCGATGAAAACGCACCAACAAAAAAATCAAAAAATTTGGCTTTGTCCTTTTTTTATCCA

TTTACGGGAAAATGTATTATTACTTTACCGCTTGAGGGAGGCTCCCACTGCCAAAGGAAA

AAAAAAGGGGGGGAGCTATCCTATTTCTAAAAAAACTTAAGCGTTGGATCCCTATTGTTA

TTTTCCCAAAGAAATTCAGAATTAATATTTGTAAACAAACCCAAAAAA

>seq21

CGCTATCGCTGACCGACGTCAGCCTTTATTAAAAAAATTGGGTTGGTGGTCGACAGGCGC

CGGCCGGCCCTAGCAAGGGCGGGTGACAAAGCCCCAAAACTGCTCGAGGACCGGACGCGG

TGCCGTCCGCTGCCTTTCGGGCCCGTCCCCGGGAAACCGGGGACGGGGGCCCAACAACAC

AAGCCGTGCTTGAGGTGCAGCAATGACGCTCGGACAGGCATGCCCCCCGGAATACCAGGG

GGCGCAATGTGCGTTCAAAGACTCGATGATTCACTGAATTCTGCAATTCACATTAGTTAT

CGCATTTCGCTGCGTTCTTCATCGATGCGGCGTTCTTCATCGATGCTGCGTTCTTTATCT

ATGCTGCGTTCTTCATCGATGCTACTTTCTTCTTCATGCTGAGTTCATGTTGAGGTCTTC

GGCGGGCGCGGGCCCAGGGGCACAAACGCCTCCGCCGCCGCCGTCCCAACACGGGGGGC

>seq22

TTAATTTTTAAAAAACCCACCTTTCCCCAGGGGAAAACCCCGCTCAAACCAGGTTTATTG

GGGGACCCCCCAAAGGGCAAGGGGGTTCAAAAAAGATATTTCATTCTCCATTCACAAAAA

ATATGGGTTTCGGTGTTTTTTTCTTTATCAAAACCAAGAGACCCATGTAAAAGTTTGATT

TTTTTTTTTTGTTGTGATTATTGTGTGGGAATTTCAAAAAAAAATATAAAAAATATTTCT

TTTTCTCTCCGGCGAACGGGGGGAGGATCATTAAGAATATTAAATATTTGTGAAATTCCA

CAGCAAACAATAATCATACAATCAAAACAAAAAAATCAAAACTTTTAACAATGGATCTCT

TGGTTCTCGATCGATGAAGAACGCAGCGAAACGCGATATTTCTTGTGAATTGCAGAAGTG

AATCATCAGTTTTTGAACGCACATTGCACTTTGGGGTATCCCCCAAAGTATACTTGTTTG

AGCGTTGTTTCTCCCTTGGAATTGCATTGCTTTTCTAAAATTTCGAATCAAATTCGTTTG

AAAAACAACACTATTCAACCTCAATCAAGTAGGATTACCCGCTGAACTTAASCCTATCAT

AAACGGAGG

>seq23

CGGGTATTCCTACCTGATCCGAGTCACATTTTAGAAGTTTGGGGTGTTTTACGGCTGTGG

CCGCGCCGCGCTCCCGGTGCGAGTGTGCAAACTACTGCGCAGGAGAGGCTGCGGCGAGAC

CGCCACTGGATTTCGGGGCCGGCACCCGTGTGAGGGCGTGCCGATCCCCAACGCCGACCC

CCCGGAGGGGTTCGAGGGTTGAAATGACGCTCGGACAGGCATGCCCGCCAGAATACTGGC

GGGCGCAATGTGCGTTCAAAGATTCGATGATTCACTGAATTCTGCAATTCACATTACTTA

TCGCATTTCGCTGCGTTCTTCATCGATGCAGCGTTC

>seq25

TGAAATATTTGTGAAATTTACACAGCAAACAATAATCATACAATCAAAACAAAAATAATC

AAAACTTTTAACAATGGATCTCTTGGTTCTCGCATCGATGAAGAACGCAGCGAAACGCGA

TATTTCTTGTGAATTGCAGAAGTGAATCATCAGTTTTTGAACGCACATTGCACTTTGGGG

TATCCCCCAAAGTATACTTGTTTGAGCGTTGTTTCTCTCTTGGAATTGCATTGCTTTTCT

AAAATTTCGAATCAAATTCGTTTGAAAAACAACACTTTCAACCTCAAATCAAGTAGGATT

ACCCGCTGAACTTACCTA

>seq26

GCTGCGGACCCGCCGCGCTCCCGATGCGAGTGTGCAAACTACTGCGCAGGAGAGGCTGCG

GCGAGACCGCCACTGTATTTCGGAGACGGCCACCCGCTAAGGGAGGGCCGATCCCCAACG

CCGACCCCCCGGAGGGGTTCGAGGGTTGAAATGACGCTCGGACAGGCATGCCCGCCAGAA

TACTGGCGGGCGCAATGTGCGTTCAAAGATTCGATGATTCACTGAATTCTGCAATTCACA

TTACTTATCGCATTTCGCTGCGTTCTTCATCGATGCA

>seq27

ACCTGCGGAAGGATCATTACCGAGCCTAGGGTTCCTAGCGAGCCAACCTCCCACCCGTGT

TTACTGTACCTTAGTTGCTTCGGCGGGCCCGCCGTCATGGCCGCCGGGGGCGTCAGCCCC

GGGCCCGCGCCCGCCGGAGACACCACGAACTCTGTCTGATCTAGTGAAGTCTGAGTTGAT

TGTATCGCAATCAGTTAAAACTTTCAACAATGGATCTCTTGGTTCCGGCATCGATGAAGA

ACGCAGCATAAAACGCAGCGG

>seq28

TGATCGAGGTAACATTCAGAATTGGGTGTTTTACGGCGTGGCCGCGCCGCTTTACAGTTG

CGAGGTGTTAGCTACTACGCAATGGAAGCTGCGGCGGGACCGCCACTGTATTTGGGGGAC

GGCGTGTGCCCGCAGGGGGCGCTCCGCCGATCCCAACGCCAGGCCCGGGGCCTGAGGGTT

GTAATGACGCTCGAACAGGCATGCCCGCCAGAATACTGGCGGGCGCAATGGCGTTCAAAG

ATTCGATGATTCACTGAATTCTGCAATTCACATTACTTATCGCATTTCGCTGCGTTCTTC

ATCGATG

>seq29

TTATTACTTGACGCTTAGCGGAAAGCGCCACTGCTTATAGGAAAGGCGGTGGAGGCGTTA

AACCGAGTAACAGGCCTGCGTAGGCTTGGTAACCTATTATAATTTACAAGAAGAGATCAG

AATTAATATTGCTACTTAACCCTAAAAAATCTATAAGGCATCGTTTAGCAACGGAACCAG

TGGTGCTCATTGCATGACCAAAGACCGCAAGTTGCGTTCAAAGACTCGATGATTCACTGA

ATATGCAATTCACACTAGTTATCGCACTTTGCTGCGTTCTTCATCGATGCA

>seq30

TCCCATCCGTGTCTATTGTACCCTGTTGCTTCGGCGGGCCCGCCGCTTGTCGGCCGCCGG

GGGGGCGCCTCTGCCCCCCGGGCCCGTGCCCGCCGGAGACCCCAACACGAACACTGTCTG

AAAGCGTGCAGTCTGAGTTGATTGAATGCAATCAGTTAAAACTTTCAACAATGGATCTCT

TGGTTCCGGCATCGATGAAGCAAGCAGCAATGAAGAACGCACCTTTGTCCAACCCACCCC

CGTTTTTTGTAATCCTTTTTTCCCGGGGCCCCCTTTCCGCGGGGGGGGGCCCCCCCCCCC

CCCCCCCCCCCCCGGGAAAAAAAAAAACCCTTTTTTTTTT

>seq31

TGCGAGGTGTTAGCTCTACCCAAGGAAAGCTGCGGCGTGCCCGCTACTGGATTTGAGGGA

CGAATAAATAAAATTTTAAAACCCAGCCGAGCCCCAACACCAGGCCCGGGGGCCTGAGGG

TTGTAATGACGCTCGAACAGGCATGCCAGCCAGAATACTGGCGGGCGCAATGTTCGTTCA

AAGCTTCGATGATTCACTGAATTCTGCATTTCACATTACTTATCGCATTTCGCTGCGTTC

TTCAT

>seq32

TGACTACTACGCTATGGAAGCTGCGGTGACCGCCAATTGAATTGGAGAAACCGGGTTACC

GCGAGTCCGAACACCAAGCTGAGCTTGAGGGATGAAAGGACGCTCGAACAGGCCTGCGCG

CCAGAATACTGGCGGCCGGCAATGTGCATTGAAAGATTCATGATTCACTGAATTCTGCAA

TTTACATTACTTATCGCATTTCGCTGCGTTTTTCATCGATGCATGCGTTCTTCATCGATG

CA

>seq40

CGATGCGGGCCCCTCGGGGCCAACCTCCCACCCGTGTTGCCCGAACCTATGTTGCCTCGG

CGGGCCCCGCGCCCGCCGACGGCCCCCCTGAACGCTGTCTGAAGTTGCAGTCTGAGACCT

ATAACGAAATTAGTTAAAACTTTCAACAACGGATCTCTTGGTTCCGGCATCGATGAAGAA

CGCAGCATCGATGAAGAACGCAGCATCAATAAAGAGCCAGCATGAATAAAGAGCCTCCAT

GAAAAACGCACCCCCCATAAAAAACGCACCCTCTATAAAAAAACCCCCC

>seq41

TCCTCCGCTTATTGATATGCTTAAGTTCAGCGGGTAATCCTACTTGATCTGAGGTTGAAT

AGTGTTGTTTTTCAAACGAATTTGATTCGAAATTTTAGAAAAGCAATGCAATTCCAAGAG

AGAAACAACGCTCAAACAAGTATACTTTGGGGGATACCCCAAAGTGCAATGTGCGTTCAA

AAACTGATGATTCACTTCTGCAATTCACAAGAAATATCGCGTTTCGCTGCGTTCTTCATC

GATGCGAGAACCAAGAGATCCATTGTTAAAAGTTTTGATTATTTTTGTTTTGACTGTAAA

ATTATTGTTTGCTGTGTAAATTTCACAAATATTATAATTCTTAATGATCCTTCCCAGGTT

CACCTACGGAGGGGAGTTTAATGAAATTTTTGTGAAAACCACACAATAATACGCAAAAAA

AATACAAAGCTTTTAACAATGGATCCCTGGTTGGACGAAAAAAA

>seq42

GGTATCCTACTTGACTGAGTTGAATTTGTAGTTGTTCTACCCACTTGAGTCCACTTTTTA

AACAAGCTTGCATGTCCTACAAAGAAACATAGCTCAACCAGGTATACTTTGGGGGATACC

CCAGAATGCCATGTGCGTTCAAAACCTGATGATTCACTTCTGCAGTTGACTGAAAAGATC

CCGTTTCGCTGCGTTCTTCATCAATGCAGGCGGGCGCAATGTGCGTTGAAAGATTTTATG

ATTCACTGAATTCTGCAATTCACATTACTTATCGCATTTTGCTGCGTTCTTCATCGATGC

ACGGCGAAACTAAGCCCTAGCGTGGACCGAAGAGGCAGGGGGGAGGATAAATTGAAAAAT

TGGGAAAATACCACCTAAAATAATATAAAATTCAAACAAAATTTAAAATTTTTCAATGGG

CTCCCCTGGTCAAAAAAAAAAAAAAACCAAAAACAAATTTTGGAAAAGAAAAAAAAAAAA

ACCCTTTTTTAAAAACCCCTTTTTTTGGGGGGGCCCCAAAGAAAAGGTGTGGAGGGGTTT

TTTCCTGGGAAAGAGTTGTGTTTTTAAAA

>seq43

GCCTCAGCGGATGCTCTGCCCCGGGTGCGTCGAAGCCCCGGTGCAAGGCGCCCGCCGGAG

AGCTGCGGCAATCTTTTTGTTGACCCCCTCACAGGTTTTTTATAATCTGAACGTTCTAGG

CCCCTCCCGTCCCCGTTTCGGAGTTGAAGGGTTGAAATGACGCTGGATCGGCTGGCTCGC

GCAAAATACTGGCAGGCGCACTGTGCGTTCAAAGATTCGATGATTCACTGAATTCTGCAA

TTCACATTACTTATCGCATTTCGCTGCGTTCTTCATCGATGCAGCGTTCTTCATCGATGC

AGCGTTCTTCATCGATGCTGCGTTCTTCATCGATGCTGCGTTCTTCAT

>seq44

AAATGAATTTATGGCACTTTTAAAAAAAAATCATATCCCCTTGTGTGCAATGTATGAGAA

AAGAGGTTGTCAAAGACCTCTTTTTTCTATATCATCAACTCTTTGCTTTGAACCAGTTTG

TCTGAAATAAATATATTCTTTGAATAACTTAATTCAAAAATACAACTTTCAACAACGGAT

CTCTTGGCTCTCGCATCGATGAAGAACGCAGCATCGATGAAGAACGCACCATCATTGAAA

AACTCAGCGAATTTGAAAAACTCATTACTTATGATAATTTGCTGCGTTCTTCATCGCTGC

TCGCGTTCTTCATCGATGCTGCGTTCTT

>seq45

AATCAGTGTTTTTATGGCACTTTCAAAATACATATAAACCTTGTGTGCAATGTCATCTCT

CTGGGGGCTGCCGGCTGTCAAAAGCCGTGTGGTCACCTTTGGGATTTATATCTACTCAGA

ACTTTAGTGATTTTGTCTGAAACATATTATGAATACTTAATTCAAAATACAACTTTCAAC

AACGGATCTCTTGGCTCTCGCATCGATGAAGAACGCAGCATCTATGAA

>seq46

CGGGTATCCTACTTGATCTGAGTTGAATAGTGTTGTTTTTAAACGAATTTGATTCGAAAT

TTTAGAAAAGCAATGCAATTCCAAGAGAGAAACAACGCTCAAACAAGTATACTTTGGGGG

ATACCCCAAAGTGCAATGTGCGTTCAAAAACTGATGATTCACTTCTGCAATTCACAAGAA

ATATCGCGTTTCGCTGCGTTCTTCATCGATGCA

>seq47

TTTACAACCTCCCAACCCCCTGTGAACATACCTAATACGTTGCCTCGGCGGATCAGCCCG

CGCCCTGTAAAAAGGGACGGCCCGCCCGAGGACCCTAAACTCTGTTTTTAGTGGAACTTC

TGAGTAAAACAAACAAATAAATCAAAACTTTCAACAACGGATCTCTTGGTTCTGGCATCG

ATGAAGAACGCAGCAAAATGGCGATAAGTAATGTGAATTGCAGAATTCAGTGAATCATCG

AATCTTTGAACGCACATTGCGCCCGCCAGTATTCTGGCGGGCATGCCTGTTCGAGCGTGC

ATTTCAAGCCCTCAAGCTCAGCTTGGTGTTGGGACTCGCGGTAACCCGCGTTCCCCAAAT

CGATTGGCGGTCACGTCGAGCTTCCATAGCGTAGTAATCATACACCTCGTTACTGGTAAT

CGTCGCGGCCACGCCGTAAAACCCCAATTTCTTGATGTGACTCGGATCAGGTAGGA

>seq48

GTGAACCTGCGGAGGGATCATTACCGAGTTTACAACTCCCCAAACCCCTGTGAACATACC

TACTTGTTGCCTCGGCGGATCAGCCCGCTCCCGGTAAAACGGGACGGCCCGCCAGAGGAC

CCCTAAACTCTGTTTCTATATGTAACTTCTGAGTAAAACCATAAATAAATCAAAACTTTC

AACAACGGATCTCTTGGTTCTGGCATCGATGAAGAACGCAGCAAAATGCGATAAGTAATG

TGAATTGCAGAATTCAGTGAATCATCGAATCTTTGAACGCACATTGCGCCCGCCAGTATT

CTGGCGGGCATGCGCGGTTCGATGCGTCATTTCAACCCTCAAGCACAGCTTGGTGTTGGG

ACTCGCGTTAATTCGCGTTCCTCAAATTGATTGGCGGTCACGTCGAGCTTCCATAGCGTA

GTAGTAAAACCCTCGTTACTGGTAATCGTCGCGGCCGCGCCGCTTAAACCCCAACTTGTG

AATGTTTACCTCGGATTCAAGTGGGAAAACCCGCCTGAAATTAAACCTATCAATAA

>seq49

TCGCCGAAGCAACGTTTAGGTATGTTCACAGGGCGGGATTGGTGAATTGAATAACTCTGT

TTTCCGTTGGTGAACCTGCGGAGGGATCATTACCGAGTTATACAACTCATCAACCCCTGT

GAACATACCTATAACGTTGCTTCGGCGGGAACAGACGGCCCCGTAACACGGGCCGCCCCC

GCCAGAGGACCCCCTAACTCTGTTTTTATAATGTTTTTTCTGAGTAAACAAGCAAATAAA

TTAAAACTTTCAACAACGGATCTCTTGGCTCTGGCATCGATGAAGAACGCAGCAAAATGC

GATAAGTAATGTGAATTGCAGAATTCAGTGAATCATCGAATCTTTGAACGCACATTGCGC

CCGCCAGTATTCTGGCGGGCATGCCTGTTCGAGCGTCATTACAACCCTCAGGCCCCCGGG

CCTGGCGTTGGGGATCGGCGGAAAAAAAAAAAAATTGGCACACGTCTTTTTTTTAAAATA

CAGTGGCACTCAAAACAGCACCAGCTTTTTTTGATCCTAGGGGGGGGGGGGGGGGAGCTG

TTCGAGCGGCGCGCCGTTACCTTAAAACTTTTTTTTTTTTTTTTTGTTCGAACGCGAAAC

AAGAAGGAAGTACTAATTCCCTCGACAAAACTTAAACATACACAGAAAAACAGAAAAAAA

CGA

>seq52

GCCCGAACCAAGTTTTCCTTTTGAAAATTTAATTTATATCCCCACTCAAATTACATTGAA

ATAAGTAAGGCCGCAAAAGGCGGTAAGTAATATGAAAGGGAGTCGATGAATAACAAATGT

TTTACGAGCCTTGGAGTGTCAATTTATGGCATGCTTTCTGTAGGTAACCTGCGGAGGGAT

CATTACCGAGTTTACAAACTAACAAACCCCTGTGTAACATACCACTGTGTTGCCTCGGCG

GTATCAGCCCGGCTCCCGTGTAAAACGGGACGGCCCGCCAGAGGACCCCTAAAAACTCTG

TTTCTATATGTAACTTCTGAGTAAAACCATAAATTAAAATCAAAACTTTCAACAACGGAT

CTCTTGGTTCTGGCATCGATGAAGAACGCAGCAAAATGCGATAAGTAATGTGAATTGCAG

AATTCAGTGAATCATCGAATCTTTGAACGCACATTGCGCCCGCCAGTATTCTGGCGGGCA

TGCCTGTTCGAGCGTCATTTCAACCCTCAAGCACAGCTTGGTGTTGGGACTCGCGTTAAT

TCGCGTTCCTCAAATTGATTGGCGGTCACGTCGAGCTTCCATAGCGTAAATAGTAAAACC

CTCGTTACTGGTAATCGTCGCGGCCACGCCGTTAAACCCCAACTTCCGTGAATGGTTGAC

TCGGRTCAG

>seq54

TTTTTATCGAAGCCAGAGCCAAGAGATCCTTTTTTTTTTTTTTATTTTTTGCTGTTAATC

AAAAAACATAATAAAAAAAGAGTTTGTTTTCTGGGGGGGCGGCCCGTTTTACAGGGCCGT

CTTTGCCGCCGAAGCAACGTATTAGGTATTTTACAGGGGGGTAGTGAGAGTTGTATAACT

CTGTTTTGCGTTGGTGAACGTGCGGAGGGATCATTACCGAGTTATACAACTCATCAACCC

TGTGAACATACTCTATATATACCAGTTGCTTCGGCGGGAACAGACGGCCCTGTAACAACG

GGCCGCCCCCGACCCAAGAGGACCCCTAACTCTGTTTTTATAATCGTTTTTCTGAGTAAA

CAAGCAAATAAATTAAAACTTTCAAGCAACGGATCTCTTGGCTCTGGCATCGATGAAGAA

CGCAGCAAAATGCGGAGTATGTAATGGGAATTGCAGAATTAAGTGAATCATCGAATCTTT

GAACGCACATTGCGCCCGCCAGTATTCTGGCGGGCATGCCTGTTCKAGCGTCATTACAAC

CCTCTAGGCCCCCGGGCCYGGAGTTGGGGATCGGCAGAAGCCCCCTGTGGGCACACGCCG

TCCCTCAAATACAGTGGCGGTCCCGCCGCAGCTTCCATTGGGGTAGTAGCTAACACCTCT

GCAACTGGAGAGCGGCGCGGCCATGCCGTAAAACACCCTTTTATGTGACTSGATGCAG

>seq55

GCCGGAACCACTTTTTTTTTTTGTTAACGTTTGATTTCCCATCGCTCAACCACCCAAATT

ATATTTTAAAAGAGAGATAGGGCGCAAAACCCCCGTAAGTAATATGAAGAGCGGAGTCCG

GCAATCAGCAAATGGATGTTCACAGGGGTGGGGAATTCAAACTTATGGCAGGCTTTTTGG

TAGGTGAACCTGCGGAGGGATCATTACCGAGTTTACAACTCACAAACCCCTGTGAACATA

CCACCTTGTTGCCTCGGCGGATCAGCCCGCTTCCCGGTAAAACGGGACGGCCCGCCAGAG

GACCCCTAAACTCTGTTTCTATATGTAACTTCTGAGTAAAACCATAAATAAATCAAAACT

TTCAACAACGGATCTCTTGGTTCTGGCATCGATGAAGAACGCAGCAAAATGCGATAAGTA

ATGTGAATTGCAGAATTCAGTGAATCATCGAATCTTTGAACGCACATTGCGCCCGCCAGT

ATTCTGGCGGGCATGCCTGTTCGAGCGTCATTTCAACCCTCAAGCACAGCTTGGTGTTGG

GACTCGCGTTAATTTCGCGTTCCTCAAATTGATTGGCGGTCACGTCGAGCTTCCATAGCG

TAATAGTAAAACCCTTGTTACTGGTAATCGTCGCGGCCACGCCGTTAAACCCCAAATTTC

CTGAATGTTGACCCGGATCAGGTAGGA
